# Multiple Origins of the Sodium Channel *kdr* Mutations in Codling Moth Populations

**DOI:** 10.1371/journal.pone.0043543

**Published:** 2012-08-17

**Authors:** Pierre Franck, Myriam Siegwart, Jerome Olivares, Jean-François Toubon, Claire Lavigne

**Affiliations:** INRA, UR1115 Plantes et Systèmes de culture Horticoles, Avignon, France; Instituto de Higiene e Medicina Tropical, Portugal

## Abstract

Resistance to insecticides is one interesting example of a rapid current evolutionary change. DNA variability in the voltage-gated sodium channel gene (trans-membrane segments 5 and 6 in domain II) was investigated in order to estimate resistance evolution to pyrethroid in codling moth populations at the World level. DNA variation among 38 sequences revealed a unique *kdr* mutation (L1014F) involved in pyrethroid resistance in this gene region, which likely resulted from several convergent substitutions. The analysis of codling moth samples from 52 apple orchards in 19 countries using a simple PCR-RFLP confirmed that this *kdr* mutation is almost worldwide distributed. The proportions of *kdr* mutation were negatively correlated with the annual temperatures in the sampled regions. Homozygous *kdr* genotypes in the French apple orchards showed lower P450 cytochrome oxidase activities than other genotypes. The most plausible interpretation of the geographic distribution of *kdr* in codling moth populations is that it has both multiple independent origins and a spreading limited by low temperature and negative interaction with the presence of alternative resistance mechanisms to pyrethroid in the populations.

## Introduction

The codling moth, *Cydia pomonella* (L.) (Lepidoptera: Tortricidae), is one of the major insect pests in the orchards (mainly apple, pear and walnut orchards), worldwide distributed in the temperate regions [1]. Chemical insecticides remain the major means used to maintain populations of this pest at a low level. As a consequence of these treatments, *C. pomonella* developed resistance to numerous insecticides in Australia [2], Americas [3], [4], [5], and Eurasia [6], [7] including resistance to synthetic pyrethroids [8], [9].

Resistance to pyrethroids is mainly conferred by modification of their primary target site: the voltage-gated sodium channel [10]. Computer-generated 3D models characterized a small number of mutations linked to insecticide-binding sites in the voltage-gated sodium channel, most of them being in the trans-membrane segments 4 to 6 of the domain II region of the protein [11]. L1014F and M918T originally found in the housefly and respectively referred to as *kdr* and *super-kdr* mutations [12] are two of the most common of these mutations in insects [13]. The *kdr* mutation is associated to moderate resistance to DTT and pyrethroids. The *super-kdr* mutation is usually linked to the *kdr* mutation and increases by tenfold primary pyrethroid resistance due to *kdr* [13], [14]. The L1014F mutation is the only voltage-gated sodium channel mutation reported so far in the codling moth [15]. It was detected in few populations over the World [8], [16], [17]. A resistance ratio of about 80-fold to the pyrethroid insecticide deltamethrin is conferred by this recessive mutation in first-instar codling moth larvae [18], [19]. A low level of pyrethroid resistance in the codling moth is also attributed to enhanced detoxification activity notably due to the P450 cytochrome oxidases [18], [20].

The evolution of resistance in insect pest populations depends on both historic and current selective processes that should be understood to manage resistance [21]. To shed light on the evolutionary processes linked to the evolution of pyrethroid resistance in *C. pomonella* populations, we analysed genetic variations at the *para* sodium channel gene. We first report countries over the World where *kdr* resistance has been observed to establish origins of resistance alleles and identify factors that may affect their global spreading. Secondly we present a more detailed analysis on populations from South-eastern France to document the impact of local pyrethroid treatments on resistance evolution at the population level.

## Materials and Methods

### Sampling

The evolution of pyrethroid resistance conferred by the sodium channel gene was investigated on codling moth samples from 52 different apple commercial orchards in 19 countries (Table 1). Codling moths were collected as diapausing larvae using corrugated cardboard traps wrapped around the trunks of apple tree. Among these 52 codling moth population samples, seven populations were previously studied [16], [17]. Codling moth populations from 21 orchards showing high larva density in South-eastern France [22] were further analysed to determine the impact of current pyrethroid treatments and interaction between resistance mechanisms on genetic variation in the *para* sodium channel gene (Table 2). Pyrethroid treatments in these French orchards encompassed mainly class II pyrethroids (esfenvalerate, fluvalinate, deltamethrine).

**Table 1:** Origins of the codling moth samples, meteorology characteristic (annual mean of the daily minimal temperature in celsius degree, annual number of freezing days) and proportions in each country of alleles (*77* and *112*) and homozygous genotypes (*77*/*77*) detected with the PCR-RFLP test [16].

Country	Year	Minimal Temperature	Freezing days	N	n	*112*	*77*	*77/77*
Armenia^a^	2002	n.a.	n.a.	30	1	0.00	1.00	1.00
Argentina^b^	2005	7.5	45	24	1	0.23	0.54	0.25
France	2006	9.1	56	771	21	0.40	0.24	0.08
New Zealand^b^	2005	8.7	5	18	1	0.61	0.17	0.00
Turkey	2010	11.8	0	42	1	0.56	0.14	0.05
USA	2008	1.3	177	217	5	0.54	0.09	0.01
Bulgaria	2007	5.3	100	60	3	0.54	0.06	0.02
Uruguay^b^	2005	11.8	0	17	1	0.32	0.06	0.00
Switzerland^a^	2003	7.2	51	35	1	0.67	0.01	0.00
Spain	2007	10.2	21	28	2	0.60	0.00	0.00
Italy	2007	7.9	11	329	5	0.36	0.00	0.00
Poland	2009	6.1	64	10	1	0.61	0.00	0.00
Czech Republic	2005	4.2	109	31	1	0.68	0.00	0.00
Greece	2006	8.9	41	20	1	0.55	0.00	0.00
Syria	2006	14.3	0	53	2	1.00	0.00	0.00
South-Africa^b^	2005	12.1	0	13	1	0.72	0.00	0.00
Morocco	2010	13.1	0	30	2	0.45	0.00	0.00
Algeria	2010	0.1	28	28	1	0.86	0.00	0.00
Chile^a^	2005	n.a.	n.a.	28	1	0.70	0.00	0.00

N and n respectively indicate the number of individuals and the number of orchards analysed per country. *77* and *77/77* respectively correspond to *kdr* allele [16] and to homozygous *kdr* genotype [18]. No meteorological data were available for the Armenian and Chilean locations (n.a.).

a These samples correspond to the population samples *A, C* and *11* that were analysed in [16].

b These samples correspond to the population samples *Ar2, NZ1, Ur2, SA1* that were analysed in [17].

**Table 2:** Proportions of alleles (*112* and *77*) and of homozygous genotypes (*77*/*77*), and expected and observed heterozygosities (H_E_/H_O_) in codling moth samples from 21 commercial apple orchards in South-eastern France.

Orchard	N	Protection	Pyrethroid treatment	Cytochrom P450 activity	*112*	*77*	*77/77*	H_E_/H_O_
154	36	Conventional	5	77	0.26	0.64	0.42	0.52/0.53
149	20	Conventional	4	687	0.43	0.38	0.10	0.66/0.60
122	37	Conventional	4	513	0.18	0.23	0.05	0.57/0.57
75	43	Conventional	4	328	0.48	0.17	0.02	0.63/0.67
65	40	Conventional	4	652	0.48	0.14	0.02	0.61/0.58
55	39	Conventional	3	635	0.45	0.24	0.10	0.65/0.56
68	21	Conventional	3	507	0.60	0.10	0.00	0.55/0.57
132	49	Conventional	3	862	0.46	0.06	0.00	0.56/0.67
35	33	Conventional	2	n.a.	0.41	0.38	0.15	0.65/0.70
84	23	Conventional	2	141	0.30	0.37	0.04	0.68/0.87
17	27	Conventional	2	542	0.30	0.35	0.11	0.56/068
140	36	Conventional	2	208	0.32	0.31	0.11	0.67/0.61
134	16	Conventional	2	340	0.31	0.16	0.00	0.62/0.81
10	29	Conventional	1	406	0.57	0.17	0.03	0.59/0.66
42	43	Conventional	1	n.a	0.52	0.14	0.02	0.60/0.56
145	41	Organic	0	188	0.28	0.45	0.22	0.65/0.63
51	59	Organic	0	32	0.38	0.25	0.05	0.66/0.66
125	52	Organic	0	70	0.41	0.23	0.04	0.66/0.71
124	45	Organic	0	90	0.39	0.17	0.02	0.63/0.76
119	58	Organic	0	34	0.45	0.16	0.03	0.62/0.57
126	24	Organic	0	265	0.40	0.13	0.04	0.61/0.54

References to crop protection practices, numbers of annual pyrethroid treatments, and cytochrom P450 oxidase activity were reported for each orchard and linked population sample. N was the number of individuals analysed per orchard. H_E_ and H_O_ were calculated with all the three alleles (*77*, *101* and *112*) detected with the PCR-RFLP. Values reported for cytochrom P450 oxidase activities were estimated as the average ECOD activity (pmol/min/individual) among the individuals collected at each orchard location. No ECOD measure was done on the individuals collected in orchards 35 and 134 (n.a.).

### Detection of the *Kdr* Mutation

Total DNA was extracted from the head of each individual following Wash *et al.* [23] with 200 µl of 10% Chelex 100 (Biorad) solution and 6 µl (10 mg/ml) of proteinase K (Eurobio). Tissues were digested over night at 56°C. After boiling for 30 minutes, supernatant was used as DNA template for PCR reaction. A PCR-RFLP test slightly modified from Franck *et al.* [16] was used to detect the *kdr* mutation. It was developed based on sequence variations in the *para* sodium channel gene of susceptible and deltamethrin resistant strains [15]. PCR amplifications were carried out with a Mastercycler thermocycler (Eppendorf) in a 25 µl reaction volume containing 1X reaction buffer (10 mM Tris-HCl, pH = 9, 50 mM KCl, 1.5 mM MgCl_2_, and 0.1 mg/ml Bovine Serum Albumin), 200 µM of each dNTPs, 0.4 µM of each *CpNa-F* and *CpNa-R* primers (Table 3), 1 unit of Go Taq DNA Polymerase (Promega) and 2 µl of DNA template. The PCR conditions were: 3 minutes at 94°C followed by 35 cycles at 94°C for 30s, 55°C for 1 min, and 72°C of elongation for 1 min with a final extension step at 72°C for 2 min. PCR products were digested at 65°C for 16 hours with 2 unit of Tsp509I endonuclease and 1X of NEB1 Buffer (New England Biolabs) in 30 µl of reaction volume. Digested products were separated by electrophoresis on 6.5% polyacrylamide denaturing gel in a Li-Cor 4200 automatic DNA sequencer. The longest digested DNA fragments in acrylamide gels were visualised using 700 IRDye labelled *CpNa-F* primer with the SAGA software (Li-Cor Biosciences). Each revealed fragment length was defined as an allele. The test was performed on 1784 individuals: 165 individuals were re-analysed according to this modified protocol [16], [17] and 1619 individuals were newly investigated (Table 1).

**Table 3:** Primers used to amplify and to sequence domain II S4-S6 region of the codling moth *para* sodium channel gene.

Primer	Sequence (5′-3′)
*SKdr-F*	GGCCGACACTTAATTTACTCATC
*SKdr-R1*	TTCCCGAAAAGTTGCATACC
*SKdr-R2*	GGGTTAACGAGCTAAACGTCCAA
*SKdr-R3*	GCAATCCCACATGCTCTCTA
*CpNa-F*	TAGAGAGCATGTGGGATTGC
*CpNa-R*	AATTTCGTAGCCCTTGATCG
*Kdr-F*	GGTGGAACTTCACCGACTTC
*Kdr-R*	GCAAGGCTAAGAAAAGGTTAAG

**Figure 1:**
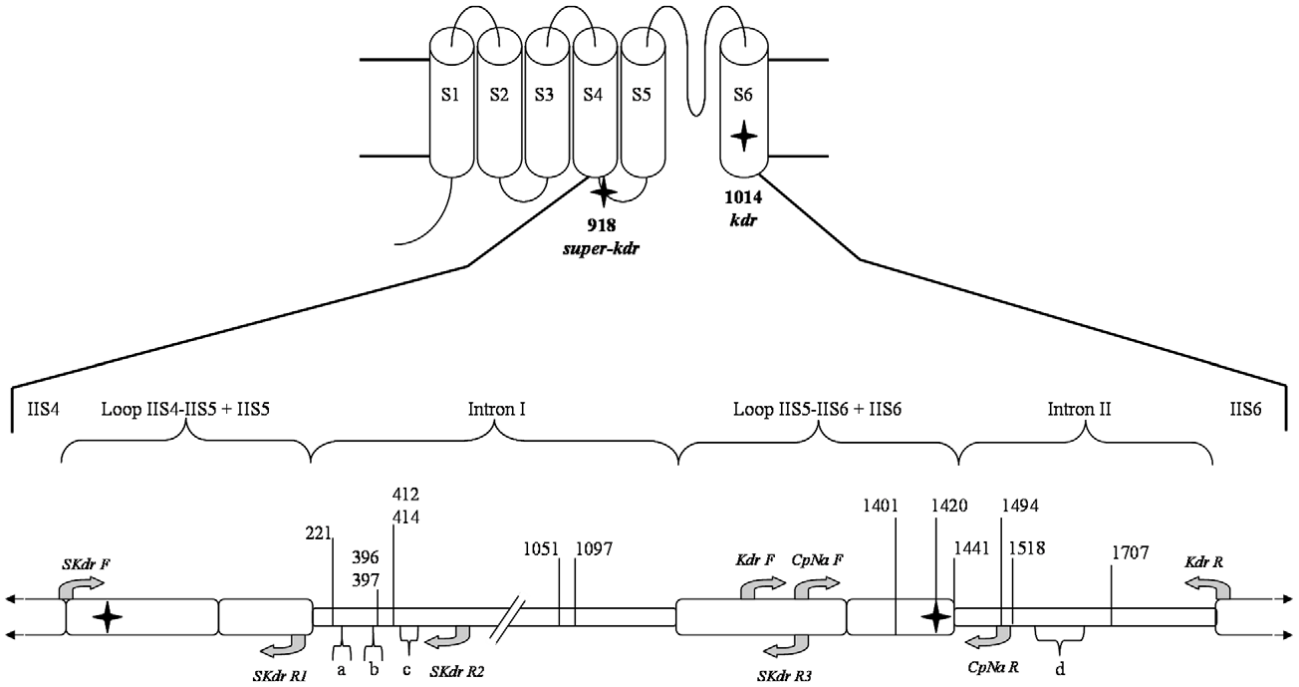
Trans-membrane segments in the domain II voltage-gated sodium channel with reference to codon position of the most frequent non-synonymous mutations and nucleotide variation in the codling moth sequences. Polymorphic sites in the nucleotide sequences are indicated as numbers (13 substitutions) or letter (4 indels) using *Syria1* sequence (1859 bp) as reference (Genbank accession number GU082334, Table 5). Arrows indicate the positions of the primers used for PCR-RFLP and sequencing analyses (Table 3).

Primer positions are indicated in Figure1. *Kdr-F* and *Kdr-R* are slightly modified from *CgD1* and *CgD2*, respectively [15]. *SKdr-R3* is the reverse of *CpNa-F.*

### Detoxification Activity by the P450 Cytochrome Oxidases

Enhanced activity of the P450 cytochrome oxidases confers heritable metabolic resistance to pyrethroid insecticides [18]. We assessed the activity of the P450 cytochrome oxidases measuring 7-ethoxycoumarin-O-deethylation (ECOD) activity on 557 moths (out of 771) collected in 19 French orchards (out of 21) to shed light on putative interaction between this resistance mechanism and pyrethroid target mutations in the sodium channel gene. ECOD activity was individually measured on abdomen samples using 0.4 mM ethoxycoumarin in 100 µL Hepes buffer [16], [24]. After four hours of incubation at 30°C, the enzymatic reaction was stopped with 100 µL of glycine buffer (10^−4^ M), pH 10.4/ethanol (v/v) and fluorescence was measured with 380 nm excitation and 465 nm emission filters on a microplate reader (HTS 7000, Perkin Elmer). ECOD activity was estimated for each moth based on the amount of 7-hydroxycoumarine formed (pg/min).

### DNA Sequencing in the *Para* Gene

DNA sequencing in the *para* gene (corresponding to trans-membrane segments 4 to 6 of the domain II region of the canal sodium protein, Figure 1) was performed on 50 codling moth individuals from various geographic origins and two *Grapholita molesta* (Lepidoptera: Tortricidae), collected in France and Brazil, to be used as an outgroup.

The whole region was sequenced for 38 codling moths displaying different homozygous genotypes according to the PCR-RFLP test (Table 4). The whole region was amplified in two independents PCRs with respectively the *SKdr-F/*S*Kdr-R3* and the *Kdr-F/Kdr-R* primer pairs (Table 3). These PCR were performed in the same conditions as above but at annealing temperatures of 56°C and 54°C respectively. The PCR products were purified from agarose gel [25] then sequenced (GATC Biotech) using, as sequencing primers, *SKdr-R1, SKdr-R2* and the four PCR primers described above (Table 3). For twelve additional codling moths collected in orchard treated with pyrethroid (Table 2), the first exon coding for the transmenbrane segments 4 and 5 was partially sequenced using the *SKdrF* and *SKdr-R1* primers in order to check for the presence of the *super-kdr* mutation.

**Table 4:** Statistical results about six generalized linear models of the proportions of *kdr* allele and of *kdr* homozygote genotypes in the orchard population samples.

Statistical models		Observed data			Statistical test		
Dependent variables	Covariables	Orchard origins	N	n	chi2	(df)	*P-*value
Proportion of *kdr* allele	Pyrethroid treatments	France	1114	19	0.16	(1)	0.687
	ECOD activity				1.00	(1)	0.317
	Pyrethroid × ECOD				0.92	(1)	0.338
Proportion of *kdr* allele	Minimal temperature	World	1830	30	5.05	(1)	0.025
Proportion of *kdr* allele	Freezing days	World	1830	30	4.57	(1)	0.033
Proportion of *kdr* homozygote	Pyrethroid treatments	France	557	19	0.09	(1)	0.768
	ECOD activity				9.16	(1)	0.003
	Pyrethroid × ECOD				2.63	(1)	0.105
Proportion of *kdr* homozygote	Minimal temperature	World	915	30	3.13	(1)	0.077
Proportion of *kdr* homozygote	Freezing days	World	915	30	3.54	(1)	0.060

N and n respectively indicate the number of individuals and the number of orchards observed for each statistical model.

### Data Analysis

The genetic variation at the sodium channel detected with the PCR-RFLP test was first analysed. In each orchard population sample, observed (H_O_) and expected (H_E_) heterozygosities were calculated and departure of genotype frequencies from Hardy-Weinberg proportions tested using the Genepop software [26] considering either all the detected alleles or only the *kdr* and the susceptible allele groups. Generalised linear models were used (*genmod* procedure, SAS version 9.1) to explain the proportions of *kdr* allele, and of homozygous *kdr* genotypes in the orchard population samples. These proportions were modelled as binomial variables with a logit link function considering the orchard as a random variable. First, the proportions of *kdr* allele or of homozygous *kdr* genotypes were modelled for the French population samples as functions of two factors (ECOD activity in moths and number of pyrethroid treatments in orchard) and their interactions (Table 2). Second, the proportions of *kdr* allele or homozygous *kdr* genotypes were modelled considering all the sampled populations that displayed *kdr* polymorphism as functions of either the annual mean of the daily minimal temperatures or the annual number of freezing days at each orchard. Meteorological data were obtained from the National Climatic Data Center website (http://www.ncdc.noaa.gov/oa/ncdc.html) for each orchard location and sampling date.

Furthermore, sequence variation at the sodium channel gene was investigated. DNA sequences were manually aligned with the Bioedit software [27]. Recombination between DNA sequences was tested using six different methods implemented in the RDP3 software [28]: RDP [29], GENECONV [30], Chimaera [31], MaxChi [31], [32], BootScan [33], and SiScan [34]. Minimum evolution trees were computed with the MEGA software [35] using a p-distance between the sequences that takes into account both substitution and indel polymorphisms. To assess the reliability of the tree, standard error tests were performed for every interior branch by resampling variable sites (1,000 bootstraps).

## Results

### Detection and Distribution of the *Kdr* Mutation in Populations

**Figure 2:**
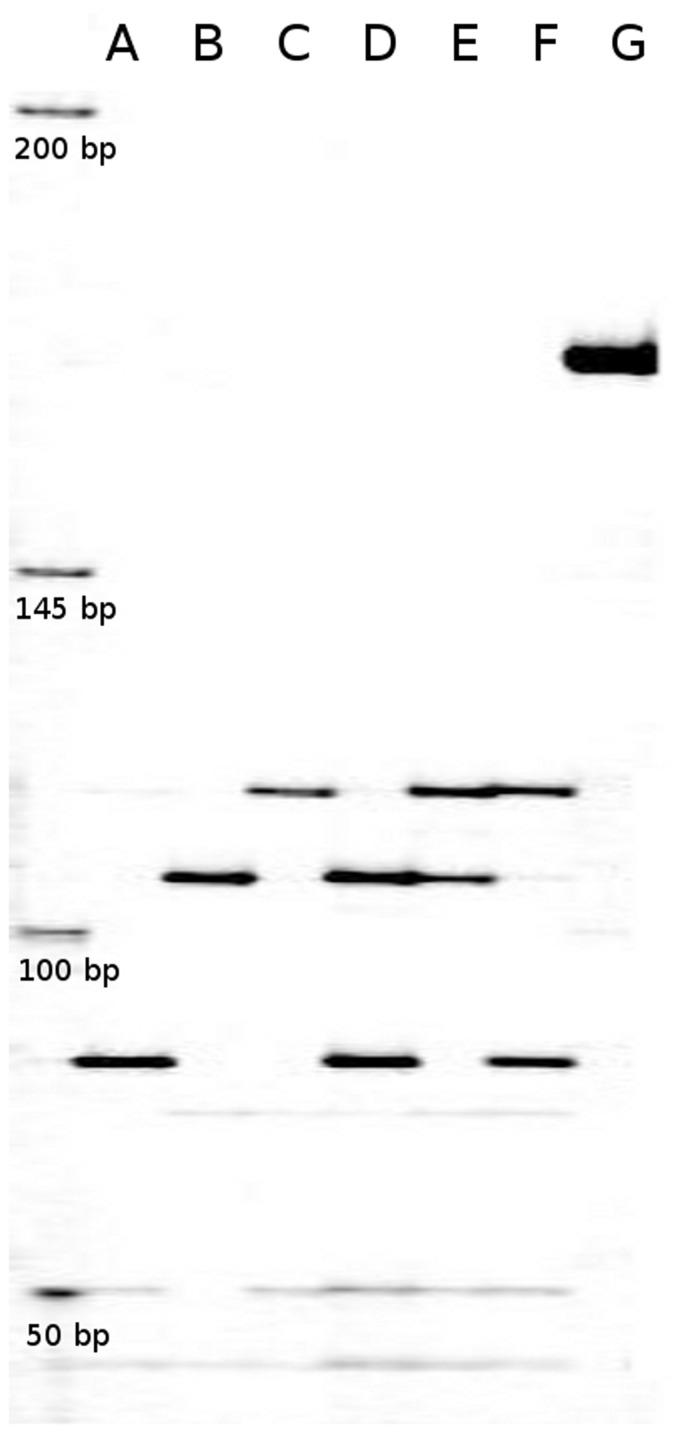
Detection of the *kdr* mutation by PCR-RFLP. PCR were conducted with the *CpNa-F* and *CpNa-R* primers (Table 3), and then digested with Tsp509I (700 IRDye labelled *CpNa-F* primer). Lengths of the restricted fragments were determined by electrophoresis in a Li-Cor 4200 automatic DNA sequencer (6.5% polyacrylamide denaturing gel) and visualised using the SAGA software (Li-Cor Biosciences). The 77 bp fragment corresponds to the *kdr* allele. The 101 and 112 bp fragments correspond to two different susceptible alleles. A–F corresponds to the different PCR-RFLP genotypes: *77/77*, *101/101*, *112/112*, *77/101*, *101/112* and *77/112*. G is the length of the non-restricted PCR product (170 bp). 50–350 pb sizing standard (Biosciences) is in the left well.

To detect the *kdr* mutation (L1014F) in codling moth populations we amplified a 170 bp region with the *CpNa-F* and *CpNa-R* primers (Figure 1 and Table 3), then digested it with the Tsp509I endonuclease that specifically cut ↓AATT sites. Two out of five restriction sites in the 170 bp region were polymorphic (Figure 1, Table 4). This polymorphism was summed up by DNA fragments of three different detectable lengths (77, 101 and 112 bp), hereafter designed as three different alleles (Figure 2). Restriction at position 1417 generated the 77 bp fragment, which was interpreted as corresponding to the *kdr* allele [16]. Restrictions at positions 1441 and 1452 in intron II respectively generated 101 and 112 bp fragments, which were interpreted as corresponding to two different susceptible alleles.

A total of 1784 codling moths were genotyped using this PCR-RFLP test (Table 1). The *77* allele was observed in population samples from all the continents except Africa and from 9 out of the 19 countries analysed. It was observed in all the samples from South-eastern France in variable proportions (Table 2). It was the only observed allele in the Armenian sample. The *101* and *112* alleles were both observed at relatively high proportions in all the other samples, except the one from Syria that was monomorphic for the *112* allele (Tables 1 and 2). No departure from Hardy-Weinberg equilibrium was detected in any sample from the 52 orchards analysed when the *101* and *112* susceptible alleles were grouped (Fisher’s exact test, *P>*0.28). Significant heterozygote excesses were detected in two French samples (Fisher’s exact test, *P = *0.03 and *P = *0.04 in the orchard sample 84 and 132, respectively) when all three alleles were considered (Table 2). The proportions of *77* allele and *77/77* genotype in the population samples from South-eastern France were slightly higher in orchards sprayed with pyrethroid insecticides (Tables 2 and 4), but these proportions did not depend on the number of pyrethroid treatments according to the generalized linear models (chi2<0.16, df = 1, *P>*0.68). A significant effect of ECOD activities was detected on the proportion of the homozygous *77/77* genotype (chi2 = 9.16, df = 1, *P = *0.0025), but not on the proportion of *77* allele (chi2 = 1.00, df = 1, *P = *0.317). Consistently, ECOD activities were lower in homozygous *77/77* genotypes (284 pg/min in average) than in other genotypes (337 pg/min in average). The proportions of the *77* allele and the *77/77* genotype were modelled according to climatic covariables using the 30 population samples over the World that displayed *kdr* polymorphism. The annual mean of the daily minimal temperatures and the annual number of freezing days in the sampled regions were highly correlated (*number of freezing days*  = −16.6 *minimal temperature* +184, R^2^ = 0.897). Consequently, generalized linear models were performed independently with these two climatic variables (Table 4). The proportions of *77* allele (chi2 = 5.05, df = 1, *P = *0.025) and, to a lesser extent, the proportions of *77/77* genotype (chi2 = 3.13, df = 1, *P = *0.077) in the codling moth samples were positively correlated with temperature in the sampled regions. Consistently, very similar negative correlations were observed with the annual number of freezing days (Table 4).

### DNA Sequencing in the Para Gene and Haplotype Divergences

**Figure 3:**
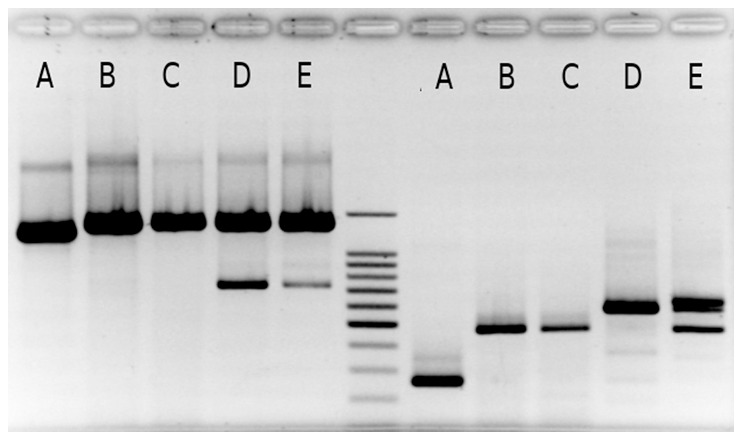
PCR product lengths obtained with the *SKdr-F/SKdr-R3* (left) and the *Kdr-F/Kdr-R* (right) primer pairs, respectively. In *Grapholita molesta*, lengths were respectively 1245 and 283 bp (A). In *Cydia pomonella*, lengths were respectively 1363 and 460 pb in specimens from France (B), 1359 and 460 pb in specimens from USA (C), 1359 and 460 or 576 pb in specimens from Syria (D and E). The electrophoresis was run on a 2% agarose gel at 100 volts for 30 minutes using the RunOne™ Electrophoresis System (Embi Technology). 100 pb DNA ladder (Promega) was in the central well.

**Figure 4:**
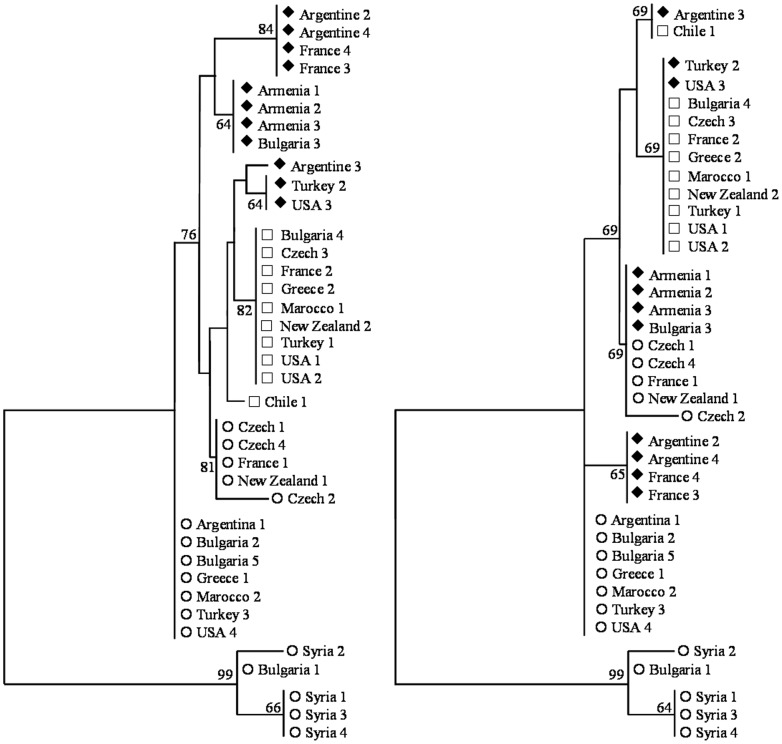
Minimum evolution trees established with the p-distance among 38 partial DNA sequences of the codling moth *para* gene. The trees were computed either with all the variable sites (left: 4 indels and 13 substitutions) or all the variable sites except the *kdr* locus (right: 4 indels and 12 substitutions). The *112* and *101* sequences (L1014) were represented by unfilled circles and squares, respectively. The *77* sequences (F1014) were represented by filled diamonds. Bootstraps values above 60% were reported.

Partial DNA sequences of the *para* gene (domain II, trans-membrane segments 5 and 6, Figure 1) were obtained for two *G. molesta* (accession number GU082359 and GU082360) and 38 *C. pomonella* individuals (GU082334-GU082358 and JQ946336–JQ946348): These 38 sequenced codling moths displayed homozygous genotypes according to the PCR-RFLP test; eleven were *77*/*77*, ten *101/101* and seventeen *112/112*. Introns I and II were both shorter in *G. molesta* than in *C. pomonella* (Figure 3) and we were not able to align and compare *G. molesta* and *C. pomonella* intron sequences. Twelve different haplotypes were identified among the 38 codling moth sequences. The sequences differed by their lengths (1649 to 1756 bp) because of the presence of three indels in intron I and one in intron II (Figure 3, Table 5). In addition, 13 substitutions were observed. Only one substitution at position 1420 was non-synonymous (L1014F). All the eleven *77*/*77* genotypes sequenced displayed at this *kdr* locus the phenylalanine amino acid. No variation was detected in the first exon that encompasses the *super-kdr* locus (50 moths sequenced including 23 displaying the *77/77* genotype). No evidence of recombination was detected among the 12 observed haplotypes with any of the six recombination tests performed with the RDP3 package. Consequently, we did not take into account recombination in the phylogenetic analyses. Thirteen out of the 17 polymorphic sites in *C. pomonella* were parsimonious informative sites. Minimum evolution trees were computed with the p-distance between the *C. pomonella* sequences using polymorphism at all the variable sites or all the variable sites except the *kdr* mutation (Figure 4). Both data sets highly supported the presence of two main clades, which differed at 11 sites in average (approximately 0.7% divergence in their nucleotide sequences). The first clade encompassed three different *112* sequences from the Bulgarian and Syrian samples. The second clade encompassed nine different *77, 101* and *112* sequences from samples distributed all over the World. Sequences within this second clade were lastly structured according to their PCR-RFLP profiles and their geographic origin. The four different *77* sequences differed from each other at three informative sites (Table 4, positions 380, 1051 and 1441) and were distributed in a least two non related subclades in addition with *101* and *112* sequences respectively (Figure 4).

**Table 5:** Sequence variability in the codling moth voltage gated sodium channel gene with variable positions numbered according to Figure 1.

Haplotype	221	265 a	380 b	396	397	412	414	447 c	1051	1097	1401	1420 *kdr*	1441	1494	1518	1528 d	1707	PCR RFLP	IntronI	IntronII
Syria 1	A	0	0	T	T	A	T	0	C	G	G	C	G	C	C	0	T	112	1106	411
Syria 2	A	0	0	T	T	A	T	−34	C	G	G	C	G	C	C	−116	T	112	1072	305
Bulgaria 1	A	0	0	T	T	A	T	0	C	G	G	C	G	C	C	−116	T	112	1106	305
Armenia 1	G	0	0	C	C	T	A	0	T	A	C	**T**	G	A	T	−116	C	77	1106	305
Turkey 1	G	0	0	C	C	T	A	0	T	A	C	C	**A**	A	T	−116	C	101	1106	305
Turkey 2	G	0	0	C	C	T	A	0	T	A	C	**T**	**A**	A	T	−116	C	77	1106	305
Czech 1	G	0	0	C	C	T	A	0	T	A	C	C	G	A	T	−116	C	112	1106	305
Czech 2	G	+7	0	C	C	T	A	0	T	A	C	C	G	A	T	−116	C	112	1113	305
Argentina 1	G	0	0	C	C	T	A	0	C	A	C	C	G	A	T	−116	C	112	1106	305
Argentina 2	G	0	+4	C	C	T	A	0	C	A	C	**T**	G	A	T	−116	C	77	1110	305
Argentina 3	G	0	0	C	C	T	A	0	C	A	C	**T**	**A**	A	T	−116	C	77	1106	305
Chile 1	G	0	0	C	C	T	A	0	C	A	C	C	**A**	A	T	−116	C	101	1106	305

Substitutions are indicated by the observed nucleotides and indels by the number of inserted or deleted base pairs using the *Syria1* sequence as reference. Nucleotides in bold characters refer to Tsp509I restriction sites. The three last columns respectively refer to lengths in base pairs of the largest Tsp509I digested fragment, and of introns I and II. Twelve different haplotypes were recognized among 38 sequences. The distribution of these twelve haplotypes among countries was reported in figure 4.

## Discussion

DNA sequences and SNPs analyses in a gene involved in insecticide resistance are complementary tools to shed light on recent evolutionary changes [13], [36]. Proximal evolutionary processes can be assessed by analysing the DNA sequence that contains the mutations involved in resistance. SNPs analyses are convenient molecular tools that allow following the dynamic of insecticide resistance in natural populations and understanding selective processes that may enhance or delay insecticide resistance evolution [21].

Although useful, SNP detection methods may fail to detect some genetic variations involved in insecticide resistance at a selected gene [37], [38]. The L1014F replacement in the voltage-gated sodium channel was primarily observed in a deltamethrin resistant codling moth strain [15]. In the present study, a rapid PCR-RFLP test was developed to monitor this *kdr* mutation in codling moth populations. However, at least two additional mutations were reported in insect pest populations at this locus: L1014S in *Culex pipiens* [39] and *Anopheles gambiae* [40] and L1014H in *Heliothis virescens* [41] and *Musca domestica* [42]. As for the F1014 variant, the S1014 but not the H1014 variants would have produced a 77 bp restriction fragment in *C. pomonella* with the developed PCR-RFLP test. However, it is unlikely that these two additional mutations are present in the codling moth: L1014F was the only non-synonymous variation in the *para* gene observed along trans-membrane segments 5 and 6 in domain II in individuals from various origins in the World. In absence of other proof, we assumed that L1014F was the only *kdr* mutation in this species.

A total of 1784 individuals collected all over the World were analysed using this PCR-RFLP test to estimate the distribution of *kdr* within and among populations. The *77* allele was observed almost worldwide and it is heterogeneously distributed among the codling moth populations. High proportions of *77/77* homozygous genotypes that are physiologically resistant to pyrethroid were only observed in Armenia, Argentina, Turkey and South-eastern France. These results confirm and extend previous observations [8], [16], [17], [43].

At the orchard level, neither *kdr* selection by current pyrethroid treatments nor *kdr* counter-selection in absence of pyrethroid treatment was evident. The proportions of *kdr* allele were not significantly correlated with the number of pyrethroid treatments in the French apple orchards. Distributions of *kdr* genotypes did not significantly depart from Hardy-Weinberg proportions in any orchard population samples. The low impact of pyrethroid treatments observed on *kdr* proportions at the orchard level seems to be a general feature whatever the within-year generation of the codling moth [44]. These results contrast with observations in *Haematobia irritans* or in *Musca domestica* populations that clearly showed seasonal variations in the proportions of *kdr* allele as a function of pyrethroid treatments [45], [46]. Three non exclusive hypotheses may explain such lack of structure of *kdr* in codling moth populations according to current insecticide applications. First, resistance management guidelines recommend alternation of pyrethroids with other insecticides among codling moth generations. Non-continuous use of pyrethroids largely limits the selection of sodium channel target mutations. This could explain why the *super-kdr* mutation is apparently absent in codling moth populations, a result also found in wild populations of horn flies [47]. Second, the usage of a large spectrum of insecticides selected various resistance mechanisms in the codling moth populations. Metabolic resistance associated with enhanced activity of the P450 cytochrome oxidases is largely spread over the World [16], [17] and confers cross-resistance to numerous insecticides including pyrethroids [8], [20]. In the present study, the activity of the P450 cytochrome oxidases was negatively correlated with the proportion of homozygous *kdr* genotypes. Metabolic resistance should be sufficient for the codling moth to resist pyrethroid treatments and it could limit selection of sodium channel target mutations in absence of strong pyrethroid selection [48]. Third, insignificant fitness cost associated with *kdr* was measured in laboratory codling moth strains [49], which could explain the maintenance of high *kdr* proportions in absence of pyrethroid selection as observed in populations from organic orchards in South-eastern France (the granulosis virus was the only insecticide used to control codling moth in these orchards). However, fitness costs are difficult to predict when several resistance mechanisms interact [50] and may depend on the environmental conditions [51].

The lack of population structure of the *kdr* genotypes detected at the orchard level contrasts with the high structure observed among populations from different orchards at larger geographic scale. First, the selection of different resistance mechanisms among countries can partially explain regional *kdr* structure. Interestingly, the Armenian populations which were the only populations that fixed the *kdr* allele in the present study were also those in which metabolic resistance conferred by the cytochrome P450 oxidases were insignificant [8], [16]. This is an additional argument supporting that *kdr* resistance evolved in interaction with other resistance mechanisms. Second, *kdr* proportions were negatively correlated with temperature in codling moth populations displaying *kdr* polymorphism in agreement with the hypothesis that fitness cost associated with sodium channel target mutations depends on temperature [51]. Consequently, fitness cost associated with *kdr* could be not equally distributed geographically and differently expressed along seasons in *C. pomonella* as previously noted in house fly populations [42], [52]. It is to note that such temperature-dependent cost could also explain the latitudinal variation in the proportion of *kdr* allele previously observed in codling moth populations from France [16]. Finally, in the absence of strong resistance cost, ancient insecticide treatments may explain current *kdr* distribution [53]. Resistance to DTT was reported in some codling moth populations in the early 1950s [54]. The first cases of resistance to pyrethroid in the 1990s in some codling moth populations from France were observed less than five years after the beginning of treatments with these insecticides [7]. DTT treatments may have initiated the selection of the *kdr* mutation in the codling moth, which was secondarily selected by pyrethroid as suggested by observations in *Plutella xylostella* [55]. Consequently, any differences among countries in DTT and pyrethroid usage during the last sixty years may also explain current differences in the proportions of *kdr* allele among codling moth populations at regional scales.

Variation in the *para* introns and exons was investigated in few insect pests – *Bemisia tabaci* [56], *Musca domestica* [42], *Mysus persicae* [57] and *Anopheles gambiiae* [40] – to shed light on the history of mutation events associated to insecticide resistance at this gene. In each species, several mutations, which likely arose independently in different populations, were linked with pyrethroid resistance. In contrast with these studies, only one substitution at the only *kdr* locus (L1014F) was observed in the codling moth. However, variations in *para* introns differentiated several haplotypes for both 1014L and 1014F variants. Phylogenetic relationships among these haplotypes confirmed the existence of two highly differentiated clades in *C. pomonella*, which is in agreement with previous analyses based on mitochondrial DNA sequences [58]. The four *kdr* haplotypes were all observed in only one clade (the most cosmopolitan one) but in several slightly differentiated sub-clades. In absence of recombination evidence, this result supports the hypothesis of independent and convergent L1014F mutation events. A subclade encompassed *kdr* haplotypes only observed in Armenia and Bulgaria, which suggests that one mutation event may have occurred somewhere in Eastern Europe. The origins of the *kdr* mutations observed in America and Western Europe remain more dubious. Among the two different *kdr* haplotypes observed in Argentina, one was identical to a French haplotype. This suggests that the *kdr* mutation in the Argentine population may have two independent origins and that one origin would be shared with the French population. This similarity supports the idea that in some situations local selection of *kdr* resistance may have followed dispersal events in *C. pomonella* as postulated to explain the unique origin of A2 esterase over-production in *Culex pipiens* [59]. Previous observations also pointed at the importance of commercial exchanges on the dispersal of codling moth larvae among continents [16]. To prevent the spread of insecticide resistance, pest control programs should think management both at local and global levels [21], [60].
